# Essential Oils and Their Constituents as Anticancer Agents: A Mechanistic View

**DOI:** 10.1155/2014/154106

**Published:** 2014-06-09

**Authors:** Nandini Gautam, Anil K. Mantha, Sunil Mittal

**Affiliations:** ^1^Centre for Environmental Science and Technology, School of Environment and Earth Sciences, Central University of Punjab, Bathinda, Punjab 151001, India; ^2^Centre for Biosciences, School of Basic and Applied Sciences, Central University of Punjab, Bathinda, Punjab 151001, India

## Abstract

Exploring natural plant products as an option to find new chemical entities as anticancer agents is one of the fastest growing areas of research. Recently, in the last decade, essential oils (EOs) have been under study for their use in cancer therapy and the present review is an attempt to collect and document the available studies indicating EOs and their constituents as anticancer agents. This review enlists nearly 130 studies of EOs from various plant species and their constituents that have been studied so far for their anticancer potential and these studies have been classified as *in vitro* and *in vivo* studies for EOs and their constituents. This review also highlights in-depth various mechanisms of action of different EOs and their constituents reported in the treatment strategies for different types of cancer. The current review indicates that EOs and their constituents act by multiple pathways and mechanisms involving apoptosis, cell cycle arrest, antimetastatic and antiangiogenic, increased levels of reactive oxygen and nitrogen species (ROS/RNS), DNA repair modulation, and others to demonstrate their antiproliferative activity in the cancer cell. The effect of EOs and their constituents on tumour suppressor proteins (p53 and Akt), transcription factors (NF-**κ**B and AP-1), MAPK-pathway, and detoxification enzymes like SOD, catalase, glutathione peroxidase, and glutathione reductase has also been discussed.

## 1. Introduction

Cancer has emerged as one of the most alarming diseases in the last few decades throughout the world. It is a multifactorial disease contributing towards uncontrolled growth and invasion of the abnormal cells leading to the formation of tumour. The steep rise in the number of cancer cases may be attributed to the change in food habits, use of tobacco and alcohol, chronic infections, exposure to harmful radiations and chemicals, or more widely due to change in lifestyle and environmental pollution [1]. International Agency for Research on Cancer (IARC) reported that there are approximately 12 million cancer cases and these have accounted for 7.6 million deaths (around 13% of all deaths) in the year 2008 [2]. The recent estimates reveal that the number of new cancer cases and cancer-related deaths has increased by 11% and 7.9, respectively, in the year 2012 as compared to 2008 [2]. Further, the developing countries have half the number of cancer incidence cases compared to the developed countries [3]. In India, 0.979 million cancer cases were reported in the year 2010 which is expected to increase to 1.148 million by 2020 [4]. The mortality rate among cancer patients is very high. The problem is more serious in economically less developed countries due to the lack of diagnostic techniques, standard methods of treatment, and higher cost of the treatment [5]. People in scientific field are currently overcoming these problems with the use of synthetic drugs. These drugs are designed to specifically target rapidly growing and dividing cells of various tumours. But, these synthetic drugs also affect rapidly dividing normal cells in our body leading to certain other major irreversible side effects. Chemotherapy used in cancer treatment has been reported to induce multidrug resistance [6, 7]. The high cost, increasing drug resistance, and side effects of current therapeutic approaches are forcing the scientists to explore alternative medicines, the traditional medicine, as an option to find new chemical entities for treatment of cancer.

Among the alternative traditional approaches, various plant products classified as alkaloids, saponins, triterpenes, glycosides, and polyphenols among others have shown very promising anticancer properties in both* in vitro* and* in vivo*. There are more than one thousand plants which have been reported to possess significant anticancer properties [8]. Vincristine, vinblastine, colchicine, ellipticine, lepachol, taxol, podophyllotoxin, camptothecin, irinotecan, etoposide, and paclitaxel are classical examples of plant-derived compounds which are found to have wide applications in cancer therapeutics [9]. The plant-derived products are expected to induce lesser side effects compared to synthetic drugs. Among plant derived compounds, essential oils (EOs) from aromatic plants have been reported to possess anticancer properties [10, 11]. EOs have also been reported to improve the quality of life of the cancer patients by lowering the level of their agony [12]. EOs-mediated therapy cannot be a substitute to the standard chemotherapy and radiotherapy but can be used in combination with cancer therapy to decrease the side effects of the drugs. Hence, EOs can be used for improving the health of the cancer patients and as a source of novel anticancer compounds. In the last two decades, a number of researches are exploring anticancer potential of EOs and their components* in vitro* and* in vivo* models. Recently, Bhalla et al. reviewed EOs as anticancer agents limiting to the recent literature and a short mechanism(s) of action [13]. However, the current review is a comprehensive one, enlisting nearly 130 studies of EOs from various plant species and their constituents that have been studied so far for their anticancer potential. The studies have been classified as* in vitro *and* in vivo* for EOs and their constituents. The current review also highlights in-depth various mechanisms of action of different EOs reported in the treatment strategies for different cancers.

## 2. Chemical Classification, Uses, and Therapeutic Potential of EOs and Their Constituents

EOs are the concentrated hydrophobic liquids with specific aroma produced by aromatic plants [14]. These are also called volatile oils or ethereal oils and are the secondary metabolites present in lower amounts in various plant parts. The composition and other biological properties of the EOs depend on their constituents. The constituents may be terpenes, aromatic compounds and some other compounds of various origins. The constituents of the EOs have been classified on the basis of their chemical structures. EOs are considered more potent than their constituents [15] due to their synergistic and more selective effect. In addition, EOs from plants growing in varied environments differ in their composition and hence have different uses. A general classification based on chemical structures along with examples is enlisted in Table 1.

EOs and their components are used for their specific aromas in perfumery and as flavouring agents in food products since ancient times. EOs have also been used in aromatherapy for improving the health due to sedative and stimulant properties. EOs are used for massage, bath, and inhalation as relaxants and treatment options as aromatherapies for various diseases with active ingredients that are being exploited in medicine [16]. The lipophilic nature of these EOs enables them to easily cross the membranes of the cells and reach inside the cell. EOs are described as strong antioxidants [10, 17] and antimicrobial [18] and are in use for the management of severe diseases like cardiovascular [19], diabetes [20], Alzheimer's [21], cancer [22], and others. However, the present review focuses only on the anticancer potential of EOs and their constituents.

## 3. EO and Constituents as Anticancer Agents

EO is one among the most valuable plant products used in the medicine and complementary treatment strategies. Exploration of EOs and their constituents toward their beneficial role in different cancers is currently under lens. A search of PubMed (http://www.pubmed.gov/), the National Institute of Health's online research shows 543 results for the search “cancer-essential oils” as of February 2014. Further screening of these research papers, nearly 135 correspond to anticancer properties of EO. Out of these 135 research papers, 117 have been published after the year 2005 indicating the sharp increase in number of publications in this field. EOs from different plants have been reported to have anticancer potential against mouth, breast, lung, prostate, liver cancer, colon cancer, and brain cancer and even in leukemia [23–28]. Not only EOs but their constituents like Carvacrol [29], d-limonene [30], Geraniols [31–33], Myrcene [34, 35], perillyl alcohol (POH) [36], **α**-humulene [37], **β**-caryophyllene [38], Thymol [39, 40], Citral [41], and others have also been reported to possess cytotoxic effect on the cancer cell lines and* in vivo* studies. Some of these like POH have gone through phase I [42] and phase II [43] clinical trials in cancer patients. Terpene analogues like Terpinen-4-ol have also been reported to have anticancer properties and induce apoptosis [44].

The current review has extensively collected and documented the available studies indicating EOs from many plants and their constituents as anticancer agents. The overall literature has been divided into different tables. Tables 2 and 3 document the* in vivo* and* in vitro* studies of EOs extracted from different plants against different cell lines along with the mechanism reported. Similarly, Table 4 documents* in vivo* and* in vitro* studies of constituents of EOs.

## 4. Mechanism of Action of EOs

**Figure 1:**
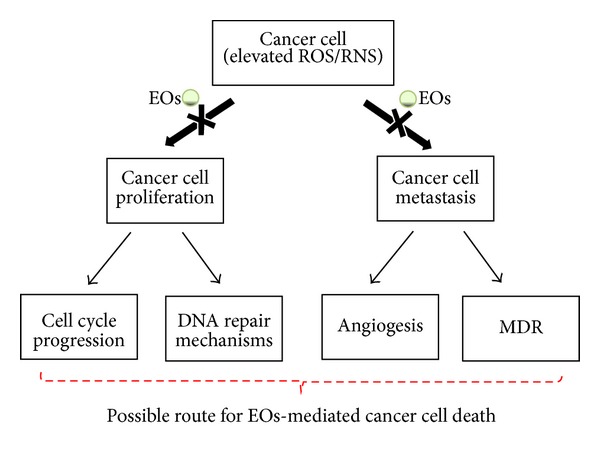
*Multitargeted role of Essential oils (EOs) towards cancer prevention*. The EOs-mediated anticancer strategies identified so far include** c**ell cycle arrest, apoptosis, and DNA repair mechanisms. EO reduces cancer cell proliferation, metastasis, and MDR which make them potential candidates toward adjuvant anticancer therapeutic agents.

Drugs used in cancer treatment target the cancer cell by inducing apoptosis or cell cycle arrest. Hence, natural products causing apoptosis in the cancer cells are valuable resources in cancer suppression. EOs with therapeutic potential can act by two ways—chemoprevention and cancer suppression. Various mechanisms involved in cancer treatment are activation of detoxification enzymes, modulation of DNA repair signaling, antimetastasis, and antiangiogenesis. Multiple pathways are involved in the antiproliferative activity demonstrated by the EOs in the cancer cells and EOs are even effective in reduction of tumours in animal models. Various targets of EOs for cancer prevention are represented in Figure 1. This makes EOs suitable anticancer agents with no large apparent effects being displayed on the normal cells. Attempts have been made to study various modes of inhibition of cancer cell growth by the EOs in this section.

### 4.1. Induction of Apoptosis

Apoptosis can occur due to effect on various signaling pathways, genetic material, and other cellular events like changes in the proteins at the intracellular level. Yu et al., using Bel-7402 cell line, had reported that the glutathione level in the body regulates cell proliferation [10]. A study on human melanoma cells reported that treatment of EOs induces DNA damage in cancer cells which is an indicator of apoptosis [45]. Apart from DNA damage, modification of various genes by the action of EOs is also responsible for apoptosis. Frank et al. studied the action of* Boswellia carteri *EO (frankincense oil) in bladder cancer cells and observed modulation of* CDKN1A*,* DEDD2*,* IER3*,* IL6*,* SGK*,* TNFAIP3 GAD45B*, and* NUDT2 genes* involved in apoptosis [46].

EOs were also demonstrated to change expression levels of Bcl-2 and* Bax* genes leading to release of cytochrome C into cytosol in KB human oral epidermoid carcinoma cells [23]. This happens via activation of caspase-9 leading to caspase-3 formation which in turn cleaves target that causes apoptosis and increased phosphorylation of extracellular signal-regulated kinase (ERK), c-jun N-terminal kinase, and p38 MAPK [23]. EO-induced apoptosis has been also suggested to be involving mitochondrial and MAPKs pathways [23]. Antiapoptotic Bcl-2 protein is downregulated by the action of EOs on the cancer cells [47]. In mouth cancer KB cells,* Artemisia lavandulaefolia* EO has been shown to decrease Bcl-2 protein level in dose dependent manner [23], which leads to apoptosis in cancer cells that is an important strategy to control cancer development and progression.

EO constituents lead to poly(ADP-ribose) polymerase-1 (PARP) cleavage [48] which is an indicator of apoptosis [49]. Major compounds of* Salvia libanotica* EO like linalyl acetate, terpineol, and camphor have been reported to be very effective against cancer. Synergistic activity of these compounds resulted in the antiproliferative effect on the isogenic colon cancer cell lines HCT-116 (p53+/+ and p53−/−) while no such effect was observed on normal intestinal cell line under similar conditions [48]. Further, Itani et al. also concluded that, in p53+/+ cells, cancer cell death occurs via mitochondrial-mediated caspase dependent pathway while in the other cells, it occurs via caspase-independent way [48]. PARP-1 protein has been shown to be modulated by the EOs and their constituents [23]. Inactivation of PARP results due to the activity of caspases leading to cancer cell death in response to treatment with EOs and their constituents. In a study,* Artemisia lavandulaefolia* EO and its major compound 1,8-cineole have been shown to adopt the above route for mitochondrial and MAPKs pathways resulting in apoptosis in the mouth cancer, KB cells [23]. EO of* Boswellia sacra* has also been reported to induce PARP cleavage in MDA-MB-231 cells [50]. Some of the mechanisms leading to apoptosis are summarised below.

#### 4.1.1. Increase in the ROS Levels

ROS are generated inside the cells in response to external stimuli or stress under normal conditions. Enhanced ROS levels in the abnormal cells instigate the cells to undergo apoptosis. Such response in the cancer cell on treatment with EO has been observed as an effective treatment method. EOs from* Aniba rosaeodora* (rosewood) were reported to induce apoptosis by increasing ROS production [51]. Similar effect has been observed by the EO of* Zanthoxylum schinifolium* in liver (HepG2) cancer cells which leads to apoptosis [52]. Decreased levels of cellular antioxidants like glutathione [53] and increased ROS production are the most commonly encountered phenomenon in cancer cells in response to the treatment with EOs that lead to cell death.

#### 4.1.2. Effect on Akt

Akt is an important protein which also regulates p53, a tumour suppressor protein.* Boswellia sacra* oil influences the Akt protein expression [50]. Vapor of* Litsea cubeba* seed oil suppressed mTOR and pPDK1 leading to dephosphorylation of Akt protein at serine (Ser^473^) and threonine (Thr^308^), respectively, activating various caspases (caspase 3 and caspase 9) which caused programmed cell death in lung cancer cells [54]. They also reported that the cell cycle gets arrested in the lung cancer cells due to overexpression of p21 resulting from the deactivation of mdm2 due to dephosphorylated Akt protein. Further increased binding of the p21 to cyclins inhibited G_1_-S phase transition [54].

#### 4.1.3. Effect on NF-*κ*B

Nuclear factor, NF-*κ*B, is a transcription factor (TF) that gets activated in the tumour cells [55]. Thus, it serves as a potential target for developing anticancer drugs and blocking of this TF advocates towards anticancer activity of the natural compounds. **α**-terpineol have been reported to target NF-*κ*B and downregulates its related genes such as* IL-1*β**,* IL1R1*,* IFNG*,* ITK*, and* EGFR* [56]. Linalyl acetate and **α**-terpineol monoterpenes act synergistically and inhibit the expression of NF-*κ*B leading to cell death of colon cancer cells [57]. Human leukaemia cell line (HL-60) treated with EO of* Cymbopogon flexuosus* and its major constituent isointermedeol has been reported to lower NF-*κ*B which is one of the contributing multiple pathways resulting in apoptosis [58]. EO of* Artemisia capillaries* leads to NF-*κ*B-DNA binding activation at the concentration above 0.5 *μ*L/mL, leading to apoptosis in the mouth cancer KB cells [47].

#### 4.1.4. Effect on AP-1

Activator protein-1 (AP-1) is another TF which plays vital role in different processes like differentiation, proliferation, transformation, and apoptosis of the cells. Its activity is regulated by MAPK proteins which are also affected by EO treatment in cancer cells [47]. Dietary intake of POH results in decreasing the tumours induced by Azoxymethane- (AOM-) induced colon cancer [59]. It prevents the skin cancer induced by UV-B radiations [60] by activation of AP-1. DNA binding activity of AP-1 increases up on effective treatment of* Artemisia capillaries* EO resulting in apoptosis in mouth cancer cells [47]. AP-1 thus is affected by the EO treatment and its activation mediate apoptosis in the cancer cells.

#### 4.1.5. MAPK-Pathway

MAP kinases get activated in response to oxidative stress in the cells [61, 62]. Various MAPKs like JNK, ERK, and p38 kinase are the signaling molecules of MAPK pathway involved in the apoptosis in cancer cell. EOs mediated apoptosis involves phosphorylated MAPK forms in the cells [62]. These forms increase with time of exposure to the EO of* Artemisia capillaris* in mouth cancer cells [47].

### 4.2. Cell Cycle Arrest

Mammalian cells have different cell cycle phases (G_1_, S, G_2_, and metaphase) to complete their life cycle. Fidelity of the cell cycle is lost due to the lack of response to the negative regulators of cell cycle progression in the cancer cells leading to uncontrolled cell division [63]. Regulation of the genes involved in this process is also hampered. Thus, halting any cell cycle event in the cancer cell leads to prevention of their growth and division, a widely employed therapeutic strategy [64]. Various cell cycle checkpoints act as potential targets for cancer treatment [64]. Patchouli alcohol which is an important component of* Pogostemon cablin* EO has been reported to upregulate p21 expression and suppress cyclin D1 and cyclin-dependent kinase 4 (CDK4) expression in colorectal cancer cells with increase in dose [65]. As p21 is negative regulator of G_1_ phase transition, increased expression of this protein by the action of patchouli alcohol is indicative of cell cycle inhibition [65]. Similar arresting of the G_1_ transition has also been reported in different types of cancer in response to various other EOs [66, 67]. EOs of* Curcuma wenyujin* inhibit S/G_2_ phase transition leading to cancer cell death [68]. G_2_/M phase transition has been reported on the treatment of liver tumour (J-5) cells with diallyl trisulfide, garlic EO constituents [69]. Various constituents like geraniol, thymol, and carvacrol of EOs inhibit different phases of cell cycle [39, 70–72]. Monoterpenes act by altering the expression of cell cycle. Genes like* DDIT3*,* IL8*, and* CDKNIA* causing cell cycle arrest have been reported to be upregulated by frankincense oil [46]. Therefore, EOs and their constituents serve as effective anticancer substances by targeting cell cycle progression in cancer cells.

### 4.3. Antimetastatic and Antiangiogenic

Angiogenesis is a process that occurs in the tumours, which helps them to survive and proliferate. Inhibition of this process stops the supply of required nutrients to the cancer cell and is an efficient way to control cancer. Certain anticancer drugs target cancer cell by this way. EO of* Curcuma zedoaria *has been tested* in vitro *and* in vivo *for antiangiogenic effect and it was reported to exhibit antiproliferative activity against various cancer cell lines and also suppressed melanoma growth and lung metastasis in mice [73]. This action was reported to be attributed towards downregulation of matrix metalloproteinases (MMP) [73]. POH which is one of the components of many EOs has been reported as the angiogenesis inhibitor molecule [74]. EO from* Citrus sinensis* has been reported to inhibit angiogenesis and metastasis in colon cancer cells [75]. Inhibition of vascular endothelial growth factor (VEGF) which plays an important role in angiogenesis is the key indicator of antiangiogenic behaviour displayed by the EOs [75]. In addition, downregulation of matrix metalloproteases (MMP-6) by the* Citrus sinensis* EO in a dose dependent manner and blockage of vascular endothelial growth factor receptor 1 (VEGFR1) also confirmed the role of EO in inhibition of metastasis in colon cancer [75]. Limonene and perillic acid are the antimetastatic molecules which are well studied in mice [76]. **α**-Pinene isolated from the EO of* Schinus terebinthifolius* also had antimetastatic activity in the C57Bl/6 mice with melanomas [77]. As both these processes are the most harmful and unique properties of the cancer cells, targeting these can prevent spreading of cancer to the other parts and inhibit proliferation of the localised tumours. Efficacy of the EOs in inhibiting these processes will enable potential treatment strategies for cancer therapy.

### 4.4. Effect on Detoxification Enzymes

Genotoxins lead to alteration of the internal antioxidants and antioxidant enzymes like superoxide dismutase (SOD), catalase (CAT), glutathione peroxidase (GPx), and glutathione reductase (GR) along with alteration of various important body functions resulting in damage to tissues and membranes. Phase I and phase II detoxification enzymes are responsible for the degradation of the harmful compounds. Certain compounds of the EOs act as inducer of these detoxification enzymes and thus prevent the induced-toxicity and even cancer in the cell line models. Citral is the example of one such compound which increases the activity of a key phase II detoxification enzyme—glutathione-S-transferase [78]. Dietary intake of (POH) also plays a role in the prevention of carcinogenesis induced by 4-(methylnitrosamino)-1-(3-pyridyl)-1-butanone [79].

EOs have been reported to have preventive effect on cancer treatment [80]. Varying concentrations of* Allium sativum* (garlic) EO were administered to mouse having diethylnitrosamine induced gastric cancer with basic diet and this EO affected phase I enzymes, SOD, CAT, and GPx activities. EO of* Allium sativum* has also been reported to be efficient in gastric cancer in mouse model [81]. EOs induce phase I and phase II enzymes which prevent the interaction of carcinogens with DNA. This results in chemopreventive effect of the EOs [15, 81].

EO of holy basil prevents fibrosarcoma tumours induced by 20-methylcholanthrene in Swiss albino mice thighs by increasing the level of endogenous antioxidants [82].* Ocimum sanctum* EO affects enzymes, namely SOD, CAT, and GST, and increases the levels of reduced glutathione, a nonenzymatic antioxidant which is responsible for decrease in the size of tumour and its incidence in the mice with the induced toxicity [82].* Salvia libanotica* EO has potential to prevent the proliferation of skin papillomas induced by 7,12 dimethylbenz[a]anthracene (DMBA)- and 12-0-tetradecanoylphorbol-13-acetate (TPA) in mice [83]. Pomegranate seed oil has ability to inhibit TPA induced skin cancer in the mice [84].

**Figure 2:**
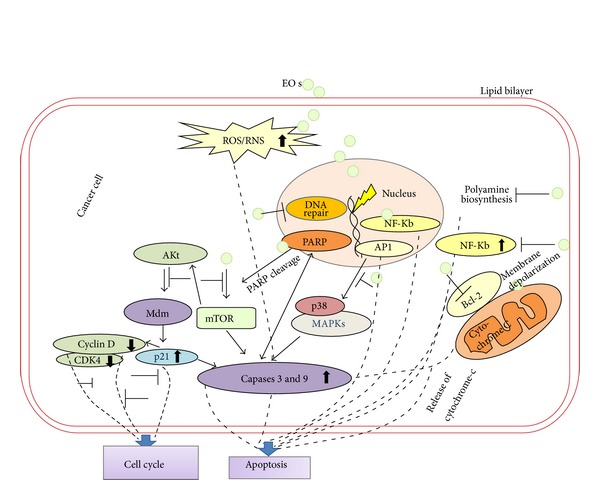
*EOs and their constituents target multiple pathways in cancer cells*. EOs by virtue have cell membrane permeability and act on different cellular targets involved in various pathways. EOs increase intracellular ROS/RNS levels which results in apoptosis in cancer cells. Inhibition of Akt, mTOR, and MAPK pathways at different steps by EOs leads to corresponding up-/downregulation of various key biomolecules (and corresponding genes which are not shown in the figure). Alteration in expression of NF-*κ*B by EOs and further binding of NF-*κ*B to DNA result in apoptosis in cancer cells. Dephosphorylation of Akt by the action of EOs results in overexpression of p21, which either induces apoptosis by increasing caspases level or results in cell cycle arrest by binding to cyclins. In addition, EOs-induced mitochondrial stress leads to activation of Bcl-2 and membrane depolarisation resulting in enhanced release of cytochrome-C to the cytoplasm which induces apoptotic cell death in cancer cells. EOs also modulate DNA repair mechanisms by acting as DNA polymerase inhibitors and lead to PARP cleavage which also results in apoptosis in cancer cells.

Antioxidant activity of EOs has been reported to be helpful in the scavenging of free radicals generated in the diseased state, leading to the cancer prevention.* Wedelia chinensis* EO has high antioxidant potential which was evaluated in lung cancer cell line implanted in C57BL/6 mice [85]. Increase in the activity of antioxidant enzymes like CAT, SOD, and GPx along with increased level of glutathione was observed in the mice model, showing the preventive effect of these EOs even in the* in vivo* models [85]. A proposed overall mechanism by which EOs display anticancer activity is presented in Figure 2.

### 4.5. Modulation of DNA Damage and Repair Signaling by EOs

Increased ROS production (as discussed above) results in DNA damage and can lead to the cell death. EOs have potential to induce damages at the DNA level that drives the cancer cells towards cell death. This activity is especially harmful in cancer cells, while no such damage is encountered in the normal cells; this provides added advantage of using these EOs. Targeting DNA repair pathways is an effective treatment method currently in use in the cancer to encounter the high proliferation rate in the cancer cells [86, 87].

One of the peculiar properties of the EOs is that though being cytotoxic to cancer cells, these induce proliferation of the normal cells [88]. DNA repair potential is present in various EOs and their constituents. Cells pretreated with the compounds like linalool, myrcene, and eucalyptol were studied for repair activity by their recovery on the normal media and it was found that these can reduce the damage caused by hydrogen peroxide (H_2_O_2_), a potential genotoxin, but their coadministration is not that beneficial [34]. Effect of the monoterpenes was dependent on the concentrations used and these had themselves induced breaks in DNA at higher concentrations [34]. Therefore, their dose response studies are important from therapeutic point of view. Camphor and thujone [89] are other monoterpenes reported to mediate via DNA repair process in the cells with induced toxicity and also known as antimutagenic in mammalian cells [89]. Thymus species EO was comparatively nontoxic to the normal fibroblast cells than MCF-7 and LNCaP human cancer cell lines [90]. IC_50_ values of* Tetraclinis articulate* EO on blood lymphocytes were reported almost double than for different cancer cells [91].

On the other hand, targeting the DNA repair pathways is helpful in cancer therapy as cells become reluctant to chemotherapy. Downregulation of the repair genes by the EOs can prove to be effective treatment strategy towards targeting DNA repair processes. Genes like* H2AFX* and* HDAC4* are responsible for DNA repair and cell cycle progression and were found to be suppressed by frankincense oil in human bladder cancer (J82) cells using microarray analysis [46]. Therefore, EOs inhibit the cancer cell progression and thereby showing anticancer properties.

More specifically, the DNA polymerases are the enzymes involved in DNA repair and replication (DNA polymerases *α*, *δ*, and *ε*). These have been reported to be very effective targets in the development of drugs for cancer treatment. EOs inhibit the activity of the DNA polymerases [11] and therefore can be used as chemotherapeutic agents in cancer treatment. Chamomile EO was found to be very strong mammalian polymerase (*λ* and *α*) inhibitor among many other EOs tested which account for their increased therapeutic potential against cancer [11]. As polymerase *α* is a DNA replicative polymerase and polymerase *λ* is a DNA repair/recombination polymerase, hence inhibition of both these polymerases will be helpful in cancer therapeutics [11].

The important DNA damage signaling protein, namely, PARP-1, is most abundantly found nuclear protein almost in all eukaryotes other than yeast. It is the first protein to act on the damaged DNA (single strand DNA and double strand DNA breaks) and initiates the DNA repair by the process of PARsylation and recruiting other DNA repair proteins associated with Base Excision Repair (BER) [86, 92] and nonhomologous end joining (NHEJ) [93]. Many EOs and their constituents lead to PARP cleavage [68]. Proteolytic cleavage of PARP-1 by the action of EOs might be indicative of modification of the DNA repair process in the cancer cells. More elaborative studies are still required in the determination of the role of EOs in modulation of different repair pathways like BER and NHEJ in cancer prevention.

## 5. Multidrug Resistance (MDR) in Cancer: A Potential Set Back

Multidrug resistance (MDR) is the most frequently encountered problem in the cancer patients, which makes most of the routinely used anticancer drugs ineffective [7, 94]. Lots of research are oriented on circumventing this problem. This arises due to different mechanisms like induction of repair of the damaged DNA in response to drug, change in drug uptake capability, and change in the level and response of the targeted enzymes. Adenosine triphosphate cassette (ABC)-transporter family proteins confer MDR due to their increased activity [95]. EOs can circumvent the reluctance of tumours to respond to the cytotoxic drugs [96]. EOs of thyme are effective against widely used drugs like Adriamycin, Vincristine, and Cisplatin resistant ovarian cancer cell lines and, in addition, tumour size reduction was also observed* in vivo* which indicates the efficacy of the EO in mammalian system [96, 97].* Juniperus excels* EO was effective against MDR P-glycoprotein-expressing CEM/ADR5000 leukemia cells and reversed their resistance indicating the use of EO in MDR treatment in cancer [98].* Melaleuca alternifolia*, tea tree oil, can ameliorate Adriamycin resistance in human melanoma cells and terpinen-1-ol is responsible for this activity [99]. Various EO constituents are reported as the anti-MDR molecules and are summarized below.

Linalool, monoterpene alcohol, is a constituent of many EOs and is reported to increase the therapeutic potential of Doxorubicin in breast cancer cells MCF-7 (adriamycin resistant) by increasing its accumulation in these cells for effective response [100]. It has also been reported to cause membrane damage in the Epirubicin-resistant lung cancer, H1299 cells [101]. Emergence of Dox resistance is the other most widely encountered chemotherapeutic hurdle [102] in the treatment of cancer patients. Thymoquinone (TQ) is the constituent of* Nigella sativa* and various other EOs have been found to prevent Dox induced resistance in breast cancer (MCF-7/Dox) cells. It inhibits their growth, induces apoptosis by upregulation of phosphatase and tensin homolog (PTEN), leading to downregulation of Akt cell survival protein, and causes cell cycle arrest at G_2_/M phase [103]. Use of the EOs as dietary supplements and coadministration with drugs can enhance the response to the treatment. However, limited studies are available in this aspect but EOs and their active constituents are the promising avenues for combating MDR in cancer patients. Hence, some EOs can be used as combinational therapy in cancer patients due to their beneficial effects after in-depth research on the capability to overcome MDR.

## 6. Prevention of Side Effects of Cancer Treatment

Cancer patients suffer from different side effects which can be preferentially reduced by alternative methods. EOs are used in the aromatherapy for reducing the agony of brain cancer patients [104]. EO is efficient in depression and reduction of anxiety in cancer patients [105]. Cancer patients undergoing chemotherapy, one of the most frequently used treatment method in cancer, are prone to various side effects [106]. These are nausea and vomiting.* Mentha spicata and M. piperita* have been found to be effective in overcoming these emetic conditions (chemotherapy-induced nausea and vomiting, CINV) along with the reduction of expenditure on treatment in the cancer patients undergoing chemotherapy [107]. EOs of* Leptospermum scoparium* and* Kunzea ericoides* were reported to prevent mucositis in the head and neck cancer patients undergoing radiotherapy when used in the preparation of mouthwash [108]. Some cancer patients having metastatic tumorigenic ulcers of skin develop necrosis and malodour [109]. Patients suffering from such malodour were reported to have improvement in their state on treatment of these ulcers with the mixture of EOs having eucalyptus, melaleuca, lemongrass, lemon, clove leaf, and thyme on a 40% ethanol base [110]. Lavender EO is widely used in aromatherapy and is found to be beneficial in reducing the distress in cancer patients [111]. Hence, EOs serve as the valuable preparations in amelioration of the side effects and sufferings of the cancer patients.

## 7. Conclusions and Future Perspectives

EOs have been used in medicine from the ancient times and the present review is an attempt to highlight their therapeutic and chemopreventive value with major emphasis on the mechanistic approaches. Main aim of summarizing the research in this area is to provide better understanding of various pathways and mode of action of different EOs. EO constituents are potent in cancer prevention and treatment. Novel potent anticancer molecules can be found in EOs which can further be exploited in therapeutics. EOs can efficiently be exploited in pharmaceutical preparations with more research and some of them are already in the different phases of clinical trials. EOs are more effective in the preliminary studies than the individual constituents. Further, EOs and their constituents can be evaluated as therapeutic agents and can be used in complementation to standard therapies. Research on EOs as anticancer therapeutic agents is still in growing stage and immense potential of the EOs needs to be explored due to the lack of target specific release. Further, studies including clinical trials are required along with the use of advanced techniques for the targeted organ-specific release of the EOs for making the treatment more effective.

## Figures and Tables

**Table 1:** Chemical classification, general formula, and structure of EO constituents with examples.

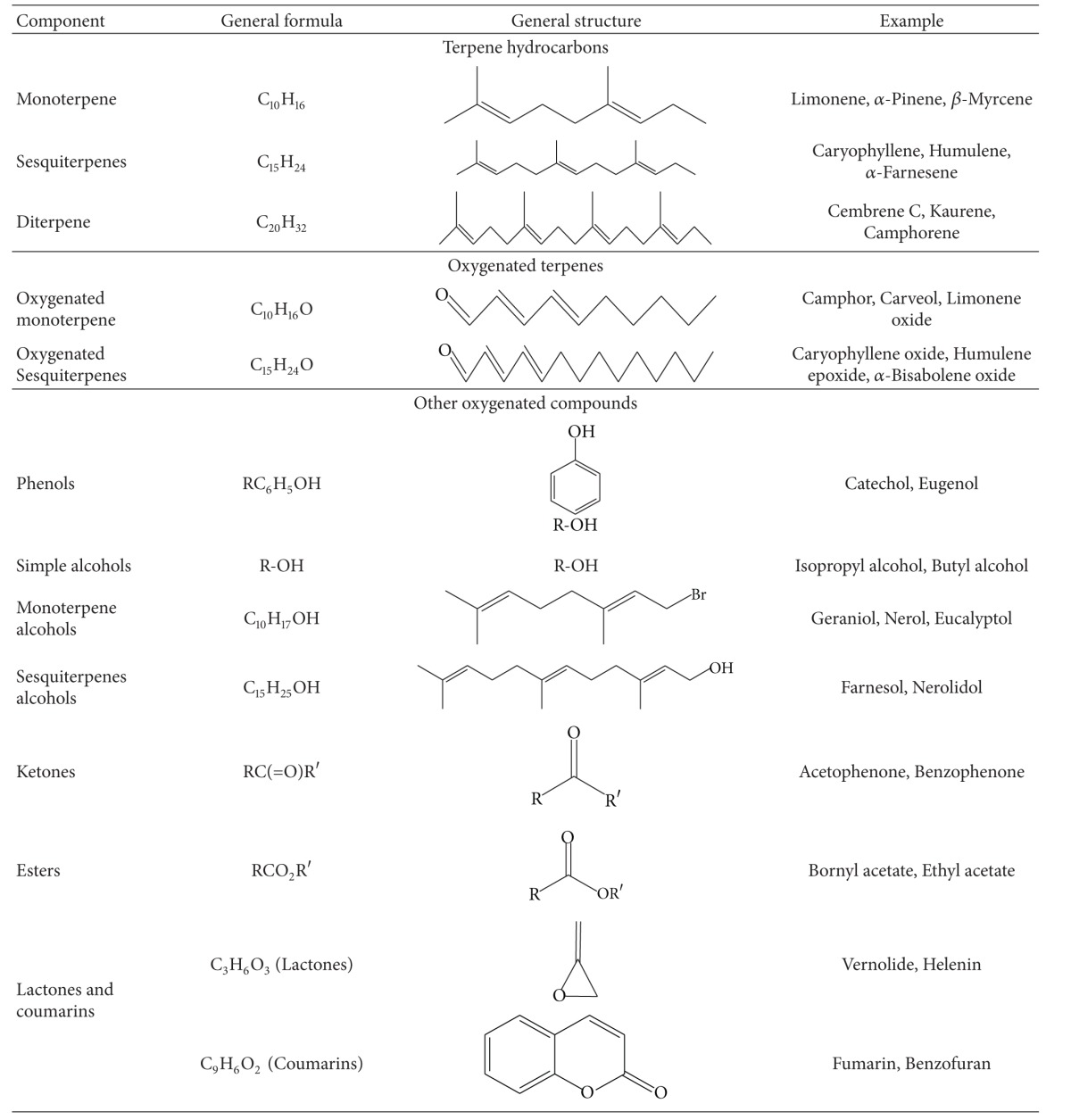

**Table 2:** List of EO bearing plants studied for anticancer potential in *in vitro* models and major observations reported.

EO bearing plants	Model system	Major findings/mechanism(s) reported	Reference
*Citrus limettioides *	Colon cancer (SW480) cells	Apoptosis via caspase-3 activation and inhibition of cox-2 and IL-6, inflammatory proteins	[24]

*Pulicaria jaubertii *	Human breast (MCF-7) and liver (HepG2) cancer cell lines	Cytotoxicity	[112]

*Drimys angustifolia *Miers and* D. brasiliensis *	Human bladder carcinoma (T24) and glioblastoma (U-138 MG) cell lines	Apoptosis	[25]

*Boswellia carterii *and* Commiphora pyracanthoides *	Human breast (MCF-7) and hepatocellular (HepG2) and cervical (HeLa), skin (HS-1) and small cell lung (A549) cancers cell lines	Cytotoxicity	[113]

*Cymbopogon citratus *and* C. nardus *	Human breast cancer (MCF-7) and non-tumorigenic (Vero) cell lines	Cytotoxicity	[114]

*Tarchonanthus camphoratus *	Human embryonic kidney and hepatocellular carcinoma cells	Cytotoxicity	[26]

*Salvia officinalis L. *	Human melanoma (A375, M14, and A2058) cell lines	Antiproliferative activity	[115]

*Thymus linearis* and *T. serpyllum *	Human breast (MCF-7), hormone dependent prostate carcinoma (LNCaP) and fibroblast (NIH-3T3) cell lines	Antiproliferative activity	[90]

*Porcelia macrocarpa *	Murine melanoma (B16F10-Nex2), human glioblastoma (U87), cervical carcinoma (HeLa), leukemia (HL-60), colon carcinoma (HCT), breast adenocarcinoma (SKBr), and melanoma (A2058); and non-tumorigenic (HFF) cell lines	Cytotoxicity	[116]

*Thymus fallax *	Human colorectal cancer (DLD-1) and mouse fibroblast (L929) cell lines	Cytotoxic to cancer but not to normal fibroblast cells	[117]

*Achillea wilhelmsii *C. Koch	Human chronic myelogenous leukemia (K562), prostate adenocarcinoma (PC3), umbilical vein endothelial (HUVEC) and cervix carcinoma (HeLa) cell lines	Cytotoxic to cancer but not to normal HUVEC cells	[118]

*Ducrosia anethifolia* and *D. flabellifolia *	Human cancer (K562, LS180 and MCF-7) cell lines	Cytotoxicity	[119]

*Xylopia frutescens *	Ovarian adenocarcinoma (OVCAR-8), bronchoalveolar lung (NCI-H358M) and metastatic prostate carcinoma (PC-3M) cell lines	Cytotoxicity	[120]

*Annona muricata *	Human breast cancer (MCF-7) cell lines	Cytotoxicity	[121]

*Lippia gracilis *	Mouse melanoma (B16-F10), human hepatocellular carcinoma (HepG2), and chronic myelocytic leukemia (K562) cell lines	G_1_ phase arrest and apoptosis via caspase-dependent pathway	[22]

*Cedrelopsis grevei *	Human breast cancer cells (MCF-7) cell lines	Cytotoxicity	[122]

*Libanotis transcaucasica *	Human cervical adenocarcinoma (HeLa), colon adenocarcinoma (LS180), breast adenocarcinoma (MCF-7) and Raji (human B lymphoma) cell lines	Cytotoxicity	[123]

*Melissa officinalis *	Human breast cancer (MCF-7) cell lines	Cytotoxicity	[124]

*Satureja intermedia *	Human oesophagus squamous cell (KYSE30) and bladder carcinoma (5637) cell lines	Cytotoxicity	[125]

*Origanum majorana *	H1299 and epirubicin-resistant H1299 cell lines	Cytotoxic and induces DNA damage	[101]

*Guatteria pogonopus *	Ovarian adenocarcinoma (OVCAR-8), bronchoalveolar lung carcinoma (NCIH358M), and metastatic prostate carcinoma (PC-3M) cell lines	Cytotoxic	[126]

*Pyrolae herba *	Human chondrosarcoma (SW1353) cells	Antitumour activity	[66]

*Thymus revolutus *	Liver cancer (HepG2) cells	Prooxidant and protective effects	[127]

*Origanum onites *L.	5RP7 (c-H-ras transformed rat embryonic fibroblasts) cell lines	Apoptosis	[128]

*Capparis spinosa* L.	Human colon carcinoma (HT-29) cell line	Inhibition of cell proliferation via G_2_/M cell cycle arrest	[129]

*Artemisia campestris* and *Thymelaea hirsuta *	Colon cancer (HT-29) cells	Antitumour activity	[27]

*Lycopus lucidus *Turcz. var. hirtus Regel	Human liver (Bel-7402 and HepG2), breast (MDA-MB-435S and ZR-75-30), cervix (HeLa) and human renal adenocarcinoma (ACHN) cell lines	Cytotoxicity	[10]

*Nigella sativa *	Human epithelial (Hep-2) cell lines	Cytotoxicity	[130]

*Thymus vulgaris *	Oral cavity squamous cell carcinoma (OCSCC) cells	Cytotoxicity	[131]

*Aniba rosaeodora *	Human epidermoid carcinoma cells (A431), epidermal keratinocytes (HEK001), normal primary epidermal keratinocytes (NHEK) and HaCaT cell lines	Apoptosis induction in selective manner	[51]

*Boswellia sacra *	Human breast cancer (T47D, MCF7, MDA-MB-231) and immortalized normal human breast (MCF10-2A) cell line	Cytotoxic to cancer cells but not to normal cells	[50]

*Syzygium aromaticum L *	Breast cancer (MCF-7 and MDA-MB-231), prostate cancer (DU-145), cervical cancer (HeLa), and Esophageal cancer (TE-13) cell lines	Cytotoxicity	[132]

*Murraya koenigii *	MCF-7, P 388, and HeLa cell lines	Antitumour activity	[133]

*Salvia officinalis *	Breast cancer (MCF-7) and colon cancer (HCT-116) cell lines	Cytotoxicity	[134]

*Mentha spicata* L., *Zingiber officinale*, *Citrus limon* Burm. f., *C*. *paradise* Macf., *Jasminum grandiflora*, lavender, *Matricaria chamomilla*, *Thymus vulgaris*, *Rosa damascena,* and cinnamon	Human prostate carcinoma (PC-3), lung carcinoma (A549), and breast cancer (MCF-7) cell lines	Cytotoxicity	[28]

*Artemisia lavandulaefolia *	Human oral epidermoid carcinoma (KB) cells	Mitochondrial stress and caspase activation mediated apoptosis	[23]

*Laurus nobilis*, *Origanum syriacum*, *Origanum vulgare*, and *Salvia triloba *	Human breast (MCF7) adenocarcinoma cells	Cytotoxicity	[135]

*Schinusmolle* L. and *Schinus terebinthifolius* Raddi	Human breast (MCF-7) cancer cells	Cytotoxicity	[136]

*Ocimum viride *	Human colorectal adenocarcinoma (COLO 205) cell line	Time and dose-dependent cytotoxicity	[137]

*Cinnamomum zeylanicum *	Normal rat embryonic fibroblasts (F2408) and c-H-ras transformed rat embryonic fibroblasts (5RP7) cell lines	Cytotoxicity and apoptosis	[138]

*Citrus reticulate*,* C. aurantium*,* C. limon*,* *and* C. aurantium *	Ehrlich ascites carcinoma resistant to Endoxan cells	Antitumour activity	[139]

*Morus rotunbiloba *Koidz	African green monkey kidney (Vero) and human larynx epidermoid carcinoma (Hep2) and colon adenocarcinoma (SW620) cell lines	Cytotoxicity	[140]

*Amomum tsaoko *	Human liver carcinoma (HepG2 and Bel-7402), cervix carcinoma (HeLa), lung carcinoma (A549), gastric adenocarcinoma (SGC-7901), prostate cancer (PC-3), hepatocyte (HL-7702), and umbilical vein endothelial (HUVEC) cell lines	Cytotoxicity to cancer cells but lesser effect on normal cell line	[141]

*Salvia pisidica *	Hepatoma G2 (HepG2) and H1299 cell lines	Protective effect against H_2_O_2_ induced toxicity	[15]

*Citrus limon *	Human cervical adenocarcinoma (HeLa) cells	Antiproliferative activity	[142]

*Rosmarinus officinalis *	Human breast cancer (MCF-7) and hormone dependent prostate carcinoma (LNCaP) cell lines	Antiproliferative activity	[143]

*Hibiscus cannabinus *	Ovarian cancer (CaOV3) and colon cancer (HT29) cell lines	Cytotoxicity and apoptosis	[144]

*Salvia rubifolia *and* S. bracteata *	Human melanoma (M14) cells	Cytotoxicity	[45]

*Croton regelianus *	Human leukemia (HL-60), melanoma (MDA-MB-435), brain (SF-295), and colon (HCT-8) cell lines	Cytotoxicity	[145]

*Citrus aurantifolia *	Colon adenocarcinoma (NIH3T3 and SW-480) cells	Apoptosis induction by DNA fragmentation and caspase-3 elevation	[146]

*Stachys cretica *ssp. *vacillans *Rech. Fil., *S*. *germanica *L., *S. hydrophila *Boiss., *S. nivea*,* S. palustris*.* *and* S. spinosa*,	Amelanotic melanoma (C32) and renal cell adenocarcinoma (ACHN) cell lines	Antiproliferative activity	[147]

*Cnidium officinale* and *Ligusticum chuanxion *	Mouse skin fibroblast (NIH 3T3) cells	Preventive effect against UVB-induced DNA damage and apoptosis	[65]

*Schefflera heptaphylla *	Breast cancer (MCF-7), melanoma (A375), and liver cancer (HepG2) cell lines	Anti-proliferative activity	[148]

*Lippia alba *	Human cervix epithelioid carcinoma cells (HeLa) and African green monkey kidney (Vero) cell lines	Cytotoxic to HeLa but not to nontumorigenic Vero	[149]

*Piper gaudichaudianum *	Chinese hamster lung fibroblast (V79 cells) cells	Cytotoxicity	[150]

*Citrus *reticulate and *Pelargonium graveolens *	Human promyelocytic leukemia (HL-60 and NB4) cell lines	Antiproliferative activity	[151]

*Boswellia *sp.	Bladder transitional cell carcinoma (J82) and normal human urothelium primary (UROtsa) cell lines	Cytotoxic to carcinoma but not normal cell line	[46]

*Salvia libanotica *	Isogenic colon cancer (HCT-116 p53+/+ and p53−/−) cell lines	Inhibitory activity	[48]

*Cinnamomum osmophloeum *	Murine macrophage (RAW 264.7) and human hepatocellular liver carcinoma (HepG2) cell lines	Cytotoxicity	[152]

*Eucalyptus sideroxylon *and *Eucalyptus torquata *	Human hepatocellular carcinoma (HepG2) and breast adenocarcinoma (MCF7) cell lines	Antiproliferative activity	[153]

*Schinus molle *	Mouse macrophage (774), mouse breast carcinoma (EMT6), mouse myoblast (C2C12) and human hepatoma (Hep3B and HepG2), bladder Carcinoma (ECV-304), and leukemic (K562) cell lines	Cytotoxicity	[154]

*Casearia sylvestris *	Human cervical carcinoma (HeLa), lung carcinoma (A549), colon adenocarcinoma (HT-29), monkey kidney (Vero) cell lines, and mice macrophages	Antitumour but less cytotoxic against Vero and Macrophages	[37]

*Curcuma wenyujin *	Human hepatoma (HepG2) cell line	Ant-proliferative activity by induction of apoptosis	[68]

*Dictamnus dasycarpus *	Human breast cancer (MCF-7, ZR-75-30 and MDA-MB-435S), liver carcinoma (Bel-7402 and HepG2), and renal adenocarcinoma (ACHN) cell lines	Antiproliferative activity with more sensitivity towards breast cancer cells	[155]

*Salvia officinalis*, *Sideritis perfoliata*, *Satureja thymbra*, *Laurus nobilis,* and *Pistacia palaestina *	Breast cancer (MCF-7), amelanotic melanoma (C32), renal cell adenocarcinoma (ACHN), and hormone dependent prostrate carcinoma (LNCaP) cell lines	Cytotoxic	[156]

*Juniperus phoenicea *	Brain tumour (U251), lung carcinoma cell line (H460), liver carcinoma cell line (HepG2), breast carcinoma cell line (MCF-7), and cervix carcinoma (HeLa)	Cytotoxic effects	[157]

*Zanthoxylum rhoifolium *Lam	Human cervical carcinoma (HeLa), lung carcinoma (A549), colon adenocarcinoma (HT-29), monkey kidney (Vero), and mice macrophages cell lines	Cytotoxic to cancer cells but not cytotoxic to Vero and Macrophage cells	[158]

*Thymus broussonetii *	Human ovarian adenocarcinoma IGR-OV1 parental OV1/P and its chemoresistant OV1/adriamycin (OV1/ADR), OV1/vincristine (OV1/VCR), and OV1/cisplatin (OV1/CDDP) cell lines	Antitumour in the cancer cells resistant to chemotherapy	[96]

*Photinia serrulata *	Human cervical carcinoma (HeLa), lung carcinoma (A-549), and liver carcinoma (Bel-7402) cell lines	Anticancer activity	[159]

*Thymus *sp.	Mastocytoma (P815) cell line	Inhibitory effect with carvacrol showing most cytotoxic	[88]

Neem* *oil	Human (MCF-7) breast cancer cell lines	Slow, nonapoptotic cell death	[160]

*Talauma ovata, Symphyopappus itatiayensis*, *Myrciaria floribunda*, *Psidium cattleianum,* and *Nectandra megapotamica *	Breast adenocarcinoma (MCF-7), colon adenocarcinoma (KM-12), multiple myeloma (RPMI-8226), prostate carcinoma (PC-3), glioblastoma (SF-268), and lung carcinoma (NCI-H460) cell lines	Cytotoxicity	[161]

*O. sanctum*,* C. citratus*, *Alpiniaofficinarum*, *L. angustifolia*,* Vetiveria zizanioides*, *Z*. *montanum*,* P. nigrum*,* C. nardus, C. longa, O. basilicum, C. hystrix, P. betel, Albizia lebbeck, O. americanum, M. spicata,* and *Psidium guajava *	Human mouth epidermal carcinoma (KB) and murine leukemia (P388) cell lines	Antiproliferative activity	[162]

*Pistacia lentiscus *var.* chia *	K562 and B16 cells	Inhibition of growth, survival, and angiogenesis	[163]

*Citrus limon, C. medica, C. sinsensis *	Human cervix carcinoma (Hela) and breast adenocarcinoma (MCF-7) cell lines	Cytotoxicity	[164]

*Eugenia caryophyllata *	Human promyelocytic leukemia cells (HL-60), histiocytic lymphoma (U-937), hepatoma (HepG2), human colon cancer (SNU-C5), and Lewis mouse lung carcinoma (3LL)	ROS mediated apoptosis	[165]

*Zanthoxylum schinifolium *	Human Hepatoma Cells (HepG2)	Apoptosis induction via ROS	[52]

*Myrica gale *	Human lung carcinoma (A549) and colon adenocarcinoma (DLD-1)	Cytotoxicity	[166]

*Abies balsamea *	MCF-7, PC-3, A549, DLD-1, M4BEU, and CT-26	Antitumour activity induced by ROS	[167]

*Lavandula stoechas *ssp.* stoechas *	Human epidermoid carcinoma (KB), human breast cancer (BC1), lung cancer (LU1), colon cancer (COL-2), drug-resistant KB (KB-V), mouse leukemia (P-388), hormone-dependent human prostate cancer (LNCaP), and rat glioma (ASK) cell lines	Variable cytotoxicity to all except ASK cell line	[168]

**Table 3:** List of EO bearing plants studied for anticancer potential in *in vivo* models and major observations reported.

EO bearing plants	*In vivo* models studied	Major findings/mechanism(s) reported	Reference
Pomegranate	Skin tumour in CD1 Mice	Chemopreventive effect	[84]

*Cymbopogon citrates* STAPF	Female Balb/C mice	Anticarcinogenic activity	[169]

*Croton regelianus *	Sarcoma 180 murine model	Antitumour activity	[145]

*Salvia libanotica *	Mice	Chemoprevention against skin papillomas	[83]

*Xylopia frutescens *	Sarcoma 180 ascites tumour cells injected in mice	Tumour growth inhibition	[120]

*Thymus broussonetii *	DBA-2/P815 (H2d) mouse model	Tumour reduction by injection of the EO	[96]

*Plectranthus amboinicus *	B16F-10 melanoma cell line injected C57BL/6 mice	Prevention of lung metastasis	[170]

*Lippia gracilis *	Sarcoma 180 bearing mice	Tumour growth inhibition	[22]

*Guatteria pogonopus *	Sarcoma 180 tumour bearing Swiss mice	Tumour inhibition	[126]

Neem	RIII/Sa female mice	Tumour reduction	[160]

*Curcuma zedoaria *	Mice	Angiogenesis inhibition	[171]

*α*-Pinene from *Schinus terebinthifolius* Raddi	C57Bl/6 mice with B16F10-Nex2 induced melanoma	Antimetastasis	[77]

**Table 4:** List of EO constituents studied for anticancer potential in both *in vitro* and *in vivo* models, and major observations reported.

Constituents used	Model systems used	Major findings/mechanism(s) reported	Reference
Azadirachtin and nimbolide	Hamster buccal pouch (HBP) carcinogenesis model	Cell cycle arrest and apoptosis by intrinsic and extrinsic pathway	[172]

Azadirachtin and nimbolide	Hepatocarcinoma (HepG2)	G_0_/G_1_ phase cell cycle and apoptosis via ROS induction and cytochrome C release in mitochondria	[173]

Azadirachtin and nimbolide	Hamster buccal pouch (HBP) carcinogenesis model	Chemoprevention of 7,12-dimethylbenz[a]anthracene (DMBA)-induced cancer, prevention of procarcinogen activation and oxidative DNA damage, upregulation of antioxidant and carcinogen detoxification enzymes, inhibition of tumour invasion and angiogenesis	[174]

Carvacrol	Male wistar albino rats with liver cancer induced by diethylnitrosamine (DEN)	Chemoprevention	[29]

Carvacrol	Human cervical cancer cell lines (HeLa and SiHa)	Apoptosis	[175]

Carvacrol	K-562, P-815, CEM, MCF-7 and MCF-7 gem (gemcitabine resistant)	Arrest in S-phase progression	[39]

Carvacrol	Liver cancer (HepG2) cell line	Apoptosis via activation of caspases and mitogen-activated protein kinase (MAPK) pathway	[176]

Carvacrol	Lung cancer (A549) cell line	Growth inhibition	[177]

Carvacrol	Human metastatic breast cancer (MDA-MB 231) cell line	Apoptosis	[178]

Carvone	Primary rat neuron and neuroblastoma (N2a) cells	Increase in antioxidant level in primary cells with little potential in treatment of brain tumour	[179]

Citral	Breast cancer (MCF-7) cell line	G_2_/M phase arrest and apoptosis	[41]

Citronellal and synthetic analog C37A (*N*-citronellylamine)	Human breast cancer (MCF-7) and a non-tumorigenic (Vero) cell line	Cytotoxicity	[114]

D-limonene	Colon cancer (LS174T) cells	Apoptosis by inactivation of *akt *pathway	[180]

Elemene	Laryngeal cancer (Hep-2) cells	Growth inhibition via decrease in eIF4E, eIF4G, bFGF and VEGF	[181]

Elemene	Colon cancer (Lovo) cells	Inhibition of telomerase activity, cell cycle arrest, and apoptosis	[182]

Eugenol	Primary melanoma (Sbcl2), radial growth phase (WM3211), primary RGP, radial and vertical growth phase (WM98-1), primary RGP and VGP, Lu-metastatic melanoma (WM1205) and Female B6D2F1 mice with B16 melanomas	Suppresses melanoma via deregulation of the E2F1 transcription factors	[183]

Eugenol	N-methyl-N′-nitro-N-nitrosoguanidine (MNNG) induced gastric cancer in rat	Tumour reduction by suppression of NF-*κ*B activation	[184]

Eugenol	Breast cancer (MCF-7) cells	Growth inhibition and apoptosis induction with decrease in levels of intracellular antioxidants	[184]

Eugenol	Androgen-insensitive prostate cancer cells (DU-145) and oral squamous carcinoma (KB) cells	Eugenol and its synthetic analogues inhibited growth without losing membrane integrity	[185]

Eugenol	Human colon cancer (HT-29) cells	Suppression of cyclooxygenase-2 activity and growth	[186]

Eugenol	Colon cancer (HCT-15 and HT-29) cells	Induction of ROS leading to apoptosis	[187]

Eugenol	Human promyelocytic leukemia (HL-60) cells	Induction of ROS, mitochondrial permeability transition (MPT), reduction of bcl-2 level, cytochrome c release leading to apoptosis	[165]

Eugenol	Human Melanoma (G361) cell line	S-phase cell cycle arrest and apoptosis	[188]

Furanodiene	Liver cancer (HepG2) cell line	G_2_/M phase arrest and apoptosis via inhibition of MAPK signalling pathway	[189]

Geraniol	Human colon cancer (Caco) cell line	Enhances sensitivity to 5-fluorouracil treatment	[32]

Geraniol	Human colon cancer (Caco) cell line	Inhibits growth and Polyamine biosynthesis-mechanism of inhibition of proliferation	[31]

Geraniol	Human colon cancer (Caco) cell line	Membrane depolarisation, decreased activity of protein kinase C activity and p44/p42 extracellular signal-regulated protein kinases (ERK)	[190]

Geraniol	Human tumours (TC-118) induced in Swiss nu/nu mice	Reduction in thymidylate synthase and thymidine kinase expression, synergistic effect of geraniol with 5-fluorouracil	[33]

Germacrene D	Murine melanoma (B16F10-Nex2), human glioblastoma (U-87 MG), cervical carcinoma (HeLa), leukemia (HL-60), colon carcinoma (HCT), breast adenocarcinoma (SKBr), and melanoma (A2058); and non-tumorigenic (HFF) cell lines	Cytotoxicity	[116]

Neem oil limonoids	Colon cancer (HCT116 p53−/−, HCT116 p21−/−) LNCaP, PPC1 and MDA-MB231 cell lines	p53 independent apoptosis autophagy	[191]

Nimbolide	Colorectal cancer (CRC) cell lines and CRC xenografts in nude mouse model	Apoptosis by caspase activation and PARP cleavage and decrease in tumour size in xenograft models	[192]

Nimbolide	Human hepatocarcinoma (HepG2)	Bcl-2, Bax, cytochrome-*c*, Smac/DIABLO, caspase-3, and caspase-9 activation leading to intrinsic pathway for apoptosis NF-*κ*B signaling	[193]

Nimbolide	Colon cancer (WiDr) cells	S-phase cell cycle arrest and caspase-mediated apoptosis	[194]

Patchouli alcohol	Human colorectal cancer (HCT 116, SW480) cells	NF-*κ*B, p 21 activation and suppression of cyclin D1 and cyclin-dependent kinase 4 (CDK4) resulting in apoptosis and decreased growth	[65]

Perillyl alcohol	Human colon carcinoma (HCT 116) cell line	Dose dependent inhibition attributed to G_1_ arrest	[70]

Perillyl alcohol	Female BALB/c mice	Tumour inhibition	[195]

Perillyl alcohol	BALB/c mice	UV-B induced AP-1 trans-activation inhibition and reduction of the tumours	[60]

Perillyl alcohol	Lung cancer (A549 and H520) cell lines	Cell cycle arrest and apoptosis	[196]

Terpinen-4-ol	Murine mesothelioma (AE17), melanoma cells (B16-F10), and fibroblasts (L929)	Necrotic cell death and apoptosis to lower extent in cancer cells	[197]

Thymol	Human promyelocytic leukemia (HL-60) cells	Caspase-dependent and independent apoptosis	[40]

Thymol	Human liver cancer (Bel-7402) cells	Antiproliferative activity	[198]

Thymol	K-562, P-815, CEM, MCF-7, and MCF-7 gem	Prevention of G_0_/G_1_ phase transition	[39]

Thymoquinone	Human colon cancer (LoVo, HCT 116, Caco-2, HT-29 and DLD-1) and human intestinal (FHs74Int) cells	ROS generation and mitogen-activated protein kinases (MAPK) JNK and ERK activation in cancer cells leading to apoptosis	[199]

Trans-caryophyllene	Breast cancer (MCF-7), colon cancer (HCT-116) and murine macrophage (RAW264.7) cell lines	Cytotoxicity	[134]

*α*-humulene	Breast cancer (MCF-7), colon cancer (HCT-116) and murine macrophage (RAW264.7) cell lines	Cytotoxicity	[134]

*α-*santalol	Breast cancer (MCF-7 and MDA-MB-231) cells	G_2_/M phase cell cycle arrest and apoptosis with little effect on normal breast cells	[200]

*β*-caryophyllene	Human tumour (MCF-7, DLD-1 and L-929) cell lines	Enhanced activity of **α**-humulene, isocaryophyllene, and paclitaxel	[38]

*β*-Caryophyllene oxide	Human prostate (PC-3) and breast cancer (MCF-7) cells	ROS generation and PI3K/AKT/mTOR/S6K1 signaling activation leading to apoptosis	[201]

*β*-elemene	Lung cancer (H460 and A549) cells	G_2_-M phase cell cycle arrest	[71]

*β*-elemene	Melanoma (B16F10) cells	Inhibition of angiogenesis via VEGF factor, antiproliferative and antimetastatic activity	[73]

*β*-elemene	Human breast cancer (MCF-7/ADM) cell line	Enhancement of adriamycin effect at its nontoxic concentration	[202]

*β*-elemene	G-422 tumour cells in mice	Cross blood brain barrier and inhibit brain carcinomas	[203]

*β*-elemene	Lung cancer (A549) cells	PI3K/Akt/mTOR/p70S6K1 signaling pathway inhibition and induces autophagy	[204]

*β*-elemene	Human ovarian cancer (A2780 and A2780/CP) cell lines	G_2_-M cell cycle arrests, cyclin B1 and Cdc2 downregulation and elevation of p53, p21waf1/cip1, p27kip1 and Gadd45 levels	[205]

*β*-elemene	Liver cancer (HepG2) cells	G_2_/M phase cell cycle arrest and apoptosis	[206]

*γ*-humulene	Colorectal cancer (HT29) cells	Apoptosis via upregulation of the CD95 receptor and CD95L on cell surface	[207]

## Abbreviations

NCBI: National Center for Biotechnology Information
IARC: International Agency for Research on Cancer
WHO: World Health Organisation
EOs: Essential oils
DNA: Deoxyribonucleic acid
PubMed: Publisher Medline
POH: Perillyl alcohol
ERK: Extracellular signal-regulated kinase
NF-*κ*B: Nuclear factor kappa-light-chain-enhancer of activated B cells
AP-1: Activator protein-1
CDK4: Cyclin-dependent kinase 4
VEGFR: Vascular endothelial growth factor receptor
VEGF: Vascular endothelial growth factor
MMP: Matrix Metalloproteases
MAPK: Mitogen-activated protein kinases
PARP: Poly(ADP-Ribose) polymerase
ROS: Reactive oxygen species
SOD: Superoxide dismutase
CAT: Catalase
GPx: Glutathione peroxidase
GR: Glutathione reductase
GST: Glutathione-S-transferase
DMBA: Dimethylbenz[A]anthracene
mTOR: Mammalian target of rapamycin
Mdm: Mouse double minute 2 homolog
p21: CDK-interacting protein 1
TPA: 12-0-Tetradecanoylphorbol-13-acetate
H_2_O_2_: Hydrogen peroxide
BER: Base excision repair
NHEJ: Nonhomologous end joining
MDR: Multidrug resistance
ABC-Transporter: Adenosine Triphosphate Cassette (ABC)-Transporter
PTEN: Phosphatase and tensin homolog
TQ: Thymoquinone.
